# Embracing diversity in dermatology: Creation of a culture of inclusion in dermatologic publishing

**DOI:** 10.1016/j.ijwd.2021.08.001

**Published:** 2021-08-05

**Authors:** Jenny E. Murase, Dedee F. Murrell

**Affiliations:** a Department of Dermatology, University of California, San Francisco, California; b Department of Dermatology, Palo Alto Foundation Medical Group, Mountain View, California; c Department of Dermatology, St. George Hospital, UNSW Faculty of Medicine, Sydney, New South Wales, Australia

Diversity in publishing is, in and of itself, a diverse concept. To be truly inclusive and embrace diversity in our dermatologic literature, it is critical that the chief editors and the editorial board of the journal examine how they are actively creating a culture of inclusion within and for their publication. Indeed, the concept of diversity includes issues regarding gender, race, and transgender/gender minority/sexual minority issues and extends beyond the medical content of what is published within the journal. With regard to the *International Journal of Women's Dermatology* (IJWD), we have taken an all-encompassing look at how to champion diversity and inclusion in our publication. Gender and racial diversity are not only limited to issues specific to women's health or care of patients with skin of color, but also include diversity in authorship as well as our editorial team, and the professional development of our female and male dermatology colleagues of different transgender/gender minority/sexual minority backgrounds. We have embraced diversity in our authorship, diversity in our colleagues, diversity in the literature, and diversity in our patients and the issues that they face regarding their dermatologic health, creating a model that can be emulated in journals throughout different fields of medicine.

## Professional development of our female and skin of color colleagues

### Diversity in our editorial and advisory boards

The editorial board of the IJWD consists of two co-editors in chief, one deputy editor, and 32 associate editors. In terms of female representation on our editorial board, we have a higher percentage of female associate editors compared with the editorial boards of all other dermatology journals. In their article, “Representation of Women as Editors in Dermatology Journals: A Comprehensive Review,” Lobl et al. (2019) demonstrated that women make up 18% of editors in chief and 22% of editorial board members. This built on previously published work in our journal regarding the low percentage of female editors-in-chief of dermatology journals (Gollins et al., 2017). This is in stark contrast to the editorial board of the IJWD, in which both of our editors-in-chief are female, 83% of our editorial board is female (29 of 35 members), and 86% of our advisory board is female (12 of 14 members).

In terms of international presence, we have 11 editors from countries outside of the United States (31% of the editorial board), including Argentina, Australia, Denmark, France, Germany, Greece, India, Iran, and Switzerland. On our advisory board, 4 of 14 members are from countries outside of the United States (29% of the advisory board), including Brazil, Germany, Israel, and the Philippines. In terms of ethnicity, we have 24 editors of Caucasian descent (67% of the editorial board), 2 editors of Black descent (6%), 2 editors of Middle Eastern descent (6%), 2 editors of Latin American descent (6%), and 5 editors of East Asian descent (14%). Finally, our advisory board also has physicians specializing in internal medicine, obstetrics and gynecology, psychiatry, and ophthalmology, providing input from within the house of medicine and guide the development of the journal so that our publications are germane to physicians within various fields of medicine who care for patients in conjunction with our dermatology colleagues.

### Authorship

As editors, we appreciate the importance of including first names in medical journals and congress publishing as a tool for assessing gender diversity in the medical literature. For many years, medicine was the prerogative of men, and physicians used titles and surnames to address each other more formally. Articles were published using surnames and initials, which makes it difficult to ascertain academic output of women. It becomes almost impossible to assess whether journals and congresses are providing sufficient opportunities for women without the use of full names. Another reason to include full names is the number of similar names in countries where there are limited surnames, such as China or Korea. Since its inception in 2015, the IJWD has been including the first names of authors to highlight the contribution of women to the articles we publish. Table 1 outlines the number of articles published per issue and the percentage of articles published with at least one female author. The impact of the IJWD on female professional colleague development is apparent in that we have published a total of 544 peer-reviewed articles to date, and an impressive 96% (522 articles) have at least one female author.

**Table 1:** Trends of Female Authorship (FA) in the *International Journal of Women's Dermatology*: Percentage of articles published with at least one female author

Year	Volume	Issue	Total articles	Articles with ≥1 female author, n	Articles with female author, %
2015	1	1	14	14	100
	1	2	12	12	100
	1	3	10	10	100
	1	4	11	10	91
2016	2	1	10	10	100
	2	2	10	10	100
	2	3	9	6	67
	2	4	10	9	90
2017	3	1	29	27	93
	3	2	10	10	100
	3	3	16	14	88
	3	4	12	12	100
2018	4	1	11	10	91
	4	2	13	12	92
	4	3	14	13	93
	4	4	27	26	96
2019	5	1	13	10	77
	5	2	15	14	93
	5	3	34	33	97
	5	4	15	15	100
	5	5	19	18	95
2020	6	1	21	20	95
	6	2	16	15	94
	6	3	21	20	95
	6	4	29	29	100
	6	5	30	30	100
2021	7	1	14	14	100
	7	2	32	32	100
	7	3	33	33	100
	7	4	34	34	100
**Total**			**544**	**522**	**96**

### Personal and professional development of female colleagues

A key initiative of both the Women's Dermatologic Society (WDS), as well as the IJWD, is to support professional female dermatologists through mentorship, leadership opportunities, and guidance regarding personal and professional development. In January 2020, Dr. Michi Shinohara, a special-issue editor for the IJWD, developed a gender gap special issue modeled after her course for the American Academy of Dermatology (Shinohara, 2020). A total of 61 articles from this special issue and over the lifetime of the journal (listed in Table 2) provide examples of publications elicited by the IJWD editors to promote female colleagues’ personal and professional development. To further this initiative, both founding co-editors in chief and current co-editors in chief, Drs. Jane Grant-Kels, Dedee Murrell, and Jenny Murase, assessed the impact of gender on mentee–mentor success through the WDS Mentorship Survey, which is now published in this September 2021 issue (Lin et al., 2021).

**Table 2:** Articles published in support of the personal and professional development of our **female colleagues** within dermatology

Sepideh Ashrafzadeh. **Gender Differences in Dermatologist Practice Locations in the U.S.: A Cross-Sectional Analysis of Current Gender Gaps.** Int J Womens Dermatol 2021; 7(4), 435-440.

Eanas Bader, Alexa R. Shipman. **The women behind the names: Dermatology eponyms named after women.** Int J Womens Dermatol 2015; 1(3), 157-160.

Wilma Bergfeld, Lynn Drake. **The Women's Dermatology Society: Physicians, Leaders, Mentors.** Int J Womens Dermatol 2015; 1(1), 2-3.

Alexander M. Cartron, Payal Shah, Jorge Roman, John Zampella. **A survey study of dermatologists' experiences of sexual harassment.** Int J Womens Dermatol 2021; 7(3), 342-343.

Alexandra Collins, Lindsay C. Strowd. **Leading ladies: Why leadership programs are so valuable to female physicians.** Int J Womens Dermatol 2020; 6(1), 54-56.

Fiona Cornish. **Mentoring and the U.K. Medical Women's Federation.** Int J Womens Dermatol 2015; 1(2), 111-112.

Benjamin S. Daniel, Dedee F. Murrell. **The role of women as past and present advocates for vaccinations: Relevance in the COVID-19 setting**. Int J Womens Dermatol 2021; 7(2), 228-229.

Adam Daunton, Alexa R. Shipman. **Professor Rona MacKie, Scottish dermatologist and melanoma authority.** Int J Womens Dermatol 2015; 1(4), 187-190.

Madeline E. DeWane, Jane M. Grant-Kels. **A commentary on gender bias in dermatology and its perceived impact on career development among women dermatologists.** Int J Womens Dermatol 2020; 6(5), 440-444.

Mytrang H. Do, Shari R. Lipner. Reply to: **Gender and rank salary trends among academic dermatologists**. Int J Womens Dermatol 2021; 7(2), 230.

Lynn A. Drake. **Dr. Wilma Fowler Bergfeld: A woman of substance, role model, and founder of the Women's Dermatologic Society.** Int J Womens Dermatol 2015; 1(3), 116-122.

Ronda S. Farah, Noah Goldfarb, Josh Tomczik, Sarah Karels, Maria K. Hordinsky. **Making the most of your mentorship: Viewpoints from a mentor and mentee.** Int J Womens Dermatol 2020; 6(1), 63-67.

Jennifer M. Gardner. **Personal productivity and the two-career household: The AIR method.** Int J Womens Dermatol 2020; 6(1), 39-42.

Jane M. Grant-Kels. **Maternity Leave for Residents and Young Attendings.** Int J Womens Dermatol 2015; 1(1), 56.

Jane Grant-Kels, Dedee Murrell. **History of Women in Dermatology series.** Int J Womens Dermatol 2015; 1(3), 115.

Jane M. Grant-Kels. **Sexism in medicine, circa 2016-2017.** Int J Womens Dermatol 2017; 3(1), 68-69.

Jane M. Grant-Kels, **The most important decision of your life is….** Int J Womens Dermatol 2017; 3(3), 184.

Jane M. Grant-Kels. **Can men mentor women in the #MeToo era?** Int J Womens Dermatol 2018; 4(3), 179.

Jane M. Grant-Kels. **Does gender impact publication opportunities?** Int J Womens Dermatol 2019; 5(2), 91.

Jane M. Grant-Kels. **Lean in or out: It is a tough balancing act, or my 10 best pieces of advice for women physicians.** Int J Womens Dermatol 2020; 6(1), 2-4.

Jane M. Grant-Kels. **Too many female doctors are part-time or stop working!?** Int J Womens Dermatol 2020; 6(1): 37-38.

Jane M. Grant-Kels. **Thoughts on dermatology residents who are new parents.** Int J Womens Dermatol 2020; 6(4), 344-345.

Jane M. Grant-Kels. **Please take your feet off my neck.** Int J Womens Dermatol 2021; 7(2), 232-233.

Jane M. Grant-Kels. **Commentary on: Sexual misconduct in academic medicine.** Int J Womens Dermatol; 2021; 7(3), 369-370.

Nava P. Greenfield. **Maternity and medical leave during residency: Time to standardize?** Int J Womens Dermatol 2015; 1(1), 55.

William D. James. **Supporting successful women in dermatology.** Int J Womens Dermatol 2020; 6(1), 70-71.

Charlotte E. Gollins, Alexa R. Shipman, Dedee F. Murrell. **A study of the number of female editors-in-chief of dermatology journals.** Int J Womens Dermatol 2017; 3(4), 185-188.

Pearl E. Grimes. **Physician burnout or joy: Rediscovering the rewards of a life in medicine.** Int J Womens Dermatol 2020; 6(1), 34-36.

Alexandra W. Hickman, Ilana S. Rosman. **Lean in or out: How to balance when the world turns upside down?** Int J Womens Dermatol 2020; 6(5), 448-449.

Faisal Khosa. **Representation of Sex, Race and Ethnicity in Pivotal Clinical Trials for Dermatologic Drugs.** Int J Womens Dermatol 2021; 7(4), 428-434.

Faisal Khosa. **Gender Disparity in Dermatologic Society Leadership: A Global Perspective.** Int J Womens Dermatol 2021; 7(4), 445-450.

A. Shadi Kourosh, Kristen M. Stewart, Molly A. Storer, Marta I. Rendon, Valerie D. Callender. **Improving young physician membership and engagement in a dermatology physician organization: The Women's Dermatologic Society as a case example.** Int J Womens Dermatol 2016; 2(1), 39-41.

Ellen J. Kim. **Working effectively with long-distance mentors.** Int J Womens Dermatol 2020; 6(1), 68-69.

Charlene Lam, Yesul Kim, Michael Cruz, Allison T. Vidimos, Elizabeth M. Billingsley, Jeffrey J. Miller. **Burnout and resiliency in Mohs surgeons: A survey study.** Int J Womens Dermatol 2021; 7(3), 319-322.

Allison R. Larson. **What “leaning in together” means to me.** Int J Womens Dermatol 2015; 1(4), 191-192.

Allison R. Larson. **Gender workforce disparities―an ethical imperative.** Int J Womens Dermatol 2019; 5(3), 177-178.

Catherine M. Ludwig, Amaris N. Geisler, Jennifer M. Fernandez, Grace Battaglia, Cathy Andorfer, Molly A. Hinshaw. **The challenge of change: Resilience traits in Women's Dermatological Society Forum participants by generation**. Int J Womens Dermatol 2020; 6(4), 277-282.

Linda Susan Marcus. **The legacy between the Women's Dermatological Society and leadership in the American Academy of Dermatology.** Int J Womens Dermatol 2019; 5(3), 192.

Sarah Mattessich, Katelyn Shea, Diane L. Whitaker-Worth. **Parenting and female dermatologists’ perceptions of work-life balance.** Int J Womens Dermatol 2017; 3(3), 127-130.
Sarah Mattessich, Anthony J. Chiaravalloti, Amy Yuntzu Yen Chen. **Dermatology residents in the era of #MeToo: Ethical considerations of appropriate responses to inappropriate patient behavior.** Int J Womens Dermatol 2019; 5(3), 175-176.

Gloria Lin, Jenny E. Murase, Dedee F Murrell, Lucas Da Cunha Godoy, Jane M. Grant-Kels **The Impact of Gender on Mentee-Mentor Success: Results from the Women's Dermatologic Society Mentorship Survey**. Int J Womens Dermatol 2021; 7(4), 398-402

Marissa Lobl, Madison Grinnell, Shauna Higgins, Kelli Yost, Pearl Grimes, Ashley Wysong. **Representation of women as editors in dermatology journals: A comprehensive review.** Int J Womens Dermatol 2019; 5(3):196-197

Mary E. Maloney, Patricia Moore. **From aggressive to assertive.** Int J Womens Dermatol 2020; 6(1), 46-49.

Dedee F. Murrell, William D. James. **Mentorship: A key mission of the Women's Dermatologic Society.** Int J Womens Dermatol 2015; 1(2), 113.

Vinod E. Nambudiri, Connie R. Shi, Ruth Ann Vleugels, Suzanne Marie Olbricht. **Academic dermatology leadership in the United States – Addressing the gender gap.** Int J Womens Dermatol 2018; 4(4), 236-237.

Eliza Notaro, Vanessa Pascoe, Michi M. Shinohara, Katherine DeNiro. **Sexual harassment from patient to provider.** Int J Womens Dermatol 2020; 6(1), 30-31.

N. Papac, Lindsey K. Collins. **The female pioneers in Mohs micrographic surgery**. Int J Womens Dermatol 2019; 5(1), 18-20.

Jodie Raffi, Megha K. Trivedi, Lucile White, Jenny E. Murase. **Work–life balance among female dermatologists.** Int J Womens Dermatol 2020; 6(1), 13-19.

Erika E. Reid, William D. James. **The history of dermatology at the Woman's Medical College of Pennsylvania.** Int J Womens Dermatol 2015; 1(2): 99-103.

Jaclyn Rosenthal, Karolyn A. Wanat, Sara Samimi. **Striving for balance: A review of female dermatologists’ perspective on managing a dual-career household.** Int J Womens Dermatol 2020; 6(1), 43-45.

Muskaan Sachdeva, Alyssa M. Thompson, Jennifer L. Hsiao, Vivian Y. Shi. Reply to commentary to: **Gender and rank salary trends among academic dermatologists.** Int J Womens Dermatol 2021; 7(2), 231.

Mona Sadeghpour, Sarah M. Sung, Heidi Jacobe, Alexa B. Kimball. **Career satisfaction of leaders in academic dermatology: Results from a national survey.** Int J Womens Dermatol 2020; 6(1), 25-29.

Kelley L. Sharp, Diane Whitaker-Worth. **Burnout of the female dermatologist: How traditional burnout reduction strategies have failed women.** Int J Womens Dermatol 2020; 6(1), 32-33.

Vivian Shi. **Gender Gaps in National Institutes of Health Dermatology Grant Recipients.** Int J Womens Dermatol 2021; 7(4), 441-444

Michi Shinohara. **The gender gap in academic dermatology and dermatology leadership: Supporting successful women dermatologists.** Int J Womens Dermatol 2020; 6(1), 1.

Aaron Steen, Kanade Shinkai. **Understanding individual and gender differences in conflict resolution: A critical leadership skill.** Int J Womens Dermatol 2020; 6(1), 50-53.

Abby S. Van Voorhees. **What will keep women practicing dermatology?** Int J Womens Dermatol 2020; 6(1): 5-6.

Ashley Wysong. **The proportion of male and female editors in women's health journals: A critical analysis and review of the sex gap.** Int J Womens Dermatol 2020; 6(1), 7-12.

Mina Yaar. **Barbara A. Gilchrest: A world-renowned dermatologist and researcher, a great mentor, an educator, former president of the Society for Investigative Dermatology, editor-in-chief of The Journal of Investigative Dermatology, and a devoted friend.** Int J Womens Dermatol 2015: 1(4), 180-186.

Rebecca Vasquez, Amit G. Pandya. **Successful mentoring of women.** Int J Womens Dermatol 2020; 6(1), 61-62.

Shali Zhang, Ha-Young Kim, Rachel E.S. Hill, Emir Veledar, Suephy C. Chen. **A ten-year comparison of women authorship in U.S. dermatology literature, 1999 vs. 2009.** Int J Womens Dermatol 2016; 2(1), 1-4.

### Personal and professional development of colleagues with skin of color

The WDS and the IJWD value diversity and inclusion in our field. To this end, we have published an array of articles specific to fostering the personal and professional development of our colleagues with skin of color within dermatology. In this September 2021 issue of the IJWD, Desai et al. (2021) discuss what it means to create a culture of equity and inclusion in dermatologic societies, mirroring our description of how to create a culture of inclusion within dermatologic publishing. The American Academy of Dermatology, WDS, American Society for Dermatologic Surgery, Association for Professors in Dermatology, American Society of Dermatopathology, American Contact Dermatitis Society, and Skin of Color Society have all taken initiatives to address the inequities in our field. It is also encouraging to see how much the twin pandemic of racism has had a positive impact on diversifying our field the past two years (Coates et al., 2021; Feaster and McMichael, 2021; Lester and Taylor, 2021; Sekyere et al., 2021). Table 3 outlines 14 articles we elicited in support of the personal and professional development of our colleagues with skin of color within dermatology.

**Table 3:** Articles published in support of the personal and professional development of our **skin of color colleagues** within dermatology

Aileen Y. Chang, Miriam Laker-Oketta, Sarah J. Coates. **Prioritizing equity and inclusion in global health dermatology.** Int J Womens Dermatol 2021; 7(2), 154-157.

Sarah J. Coates, Aileen Y. Chang, Jenna C. Lester. **Responding to the moment: Dermatology, health inequity, and a call for introspection-driven activism.** Int J Womens Dermatol 2021; 7(2), 187-188.

Seemal R Desai, Rayva Khanna, Donald Glass, Murad Alam, Vicky Barrio, Lars E French, Mona Gohara,, Lynn McKinley-Grant, Valerie Harvey, Candrice Heath, Kavita Mariwalla, Alice Pentland, Melissa Piliang, Crystal Pourciau, Susan Taylor, Peggy Wu, Pearl Grimes, Henry W. Lim. **Embracing Diversity in Dermatology: Creation of a Culture of Equity and Inclusion in Dermatology**. Int J Womens Dermatology 2021; 7(4), 378-382

Brittany Feaster, Amy J. McMichael. **Allyship in dermatology.** Int J Womens Dermatol 2021; 7(2), 152-153.

Nasro A. Isaq, Sacharitha Bowers, Steven T. Chen. **Taking a “step” toward diversity in dermatology: De-emphasizing USMLE Step 1 scores in residency applications.** Int J Womens Dermatol 2020; 6(3), 209-210.

Jenna C. Lester, Susan C. Taylor. **Two pandemics: Opportunities for diversity, equity and inclusion in dermatology.** Int J Womens Dermatol 2021; 7(2), 137-138.

Jenny E. Murase, Dedee F. Murrell. **Heralding change within dermatology: Response of the International Journal of Women's Dermatology (IJWD) to the twin pandemic of racism.** Int J Dermatol 2021; 7(2), 125-126.

Ginette A. Okoye. **Supporting underrepresented minority women in academic dermatology**. Int J Womens Dermatol 2020; 6(1): 57-60.

Ginikanwa Onyekaba, Ademide Adelekun, Susan C. Taylor, Temitayo Ogunleye. **Assessing the impact of an intervention to increase minority representation in dermatology.** Int J Womens Dermatol 2021; 7(2), 199-200.

Victoria Perez, Mona Gohara. **If you want to be it, it helps to see it: Examining the need for diversity in dermatology.** Int J Womens Dermatol 2020; 6(3), 206-208.

Katherine L. Perlman, Natalie M. Williams, Ista A. Egbeto, David X. Gao, Naomi Siddiquee, Joyce H. Park. **Skin of color lacks representation in medical student resources: A cross-sectional study.** Int J Womens Dermatol 2021; 7(2), 195-196.

Nana Amma N. Sekyere, Pearl E. Grimes, Wendy E. Roberts, Valerie D. Callender, Lenore Kakita, Jenny Murase. **Turning the Tide: How the Women's Dermatologic Society Leads in Diversifying Dermatology.** Int J Womens Dermatol 2021; 7(2), 135-136.

Claire Stewart, Shari R. Lipner. **Gender and race trends in academic rank of dermatologists at top U.S. institutions: A cross-sectional study**. Int J Womens Dermatol 2020; 6(4), 283-285.

Alison Tran, Mona Gohara. **Community engagement matters: A call for greater advocacy in dermatology**. Int J Womens Dermatol 2021; 7(2), 189-190.

### Skin of color and diversity and inclusion in our literature

As co-editors in chief, we have embarked on initiatives to both increase the skin-of-color content of our publication and create a baseline metric for how dermatology journal skin-of-color content can be tracked over time. These two strategic initiatives were performed with the intention of promoting a culture of inclusion within our publication. The March 2021 issue of the IJWD featured 24 invited publications written on topics ranging from diversity within dermatology, allyship and advocacy, and diseases directly related to pigmentation and high hair curl type prevalent in skin-of-color populations (Murase and Murrell, 2021). In this September 2021 issue, we feature a study in which we developed a two-tier criterion to assess skin-of-color content for 52 dermatologic publications and ranked each of the dermatologic publications by their skin-of-color content, analyzing the most common categorizations and differences based on Scopus score, international versus non-international, and scientific versus clinical publications (Wilson et al., 2021a; 2021b). These initiatives sent a clear message to our authorship, our readership, and our editorial board that we are interested in publishing articles on skin of color and diversity issues (52 articles are provided as examples in Table 4), which further helps to build a culture of inclusion, both within the editorial board and for the public facing aspects of our journal.

**Table 4:** Articles published in support of dermatologic health concerns for our **skin of color** patients

Ladan Afifi, Lina Saeed, Lauri A. Pasch, Heather Gibson Huddleston, Marcelle I. Cedars, Lee Thomas Zane, Kanade Shinkai. **Association of ethnicity, Fitzpatrick skin type, and hirsutism: A retrospective cross-sectional study of women with polycystic ovarian syndrome.** Int J Womens Dermatol 2017; 3(1), 37-43.

Akachi Agor, Kimberley H.M. Ward. **Camouflaging techniques for patients with central centrifugal cicatricial alopecia.** Int J Womens Dermatol 2021; 7(2), 180-183.

Mohamed A. Al-Kamel. **Impact of leishmaniasis in women: a practical review with an update on my ISD-supported initiative to combat leishmaniasis in Yemen (ELYP).** Int J Womens Dermatol 2016; 2(3), 93-101.

Mina Almasi-Nasrabadi, Reza M. Robati, Omid Zargari, Mohammad Shahidi-Dadras. **Considerable variation among Iranian dermatologists in the knowledge and attitudes regarding the use of biologic agents to manage psoriasis.** Int J Womens Dermatol 2019; 5(5), 356-360.

Reema Ruddah Almuqati, Ali Saeed Alamri, Nawal Ruddah Almuqati. **Knowledge, attitude, and practices toward sun exposure and use of sun protection among non-medical, female, university students in Saudi Arabia: A cross-sectional study.** Int J Womens Dermatol 2019; 5(2), 105-109.

Saad F. Alrayyes, Sarah F. Alrayyes, Umar D. Farooq. **Skin-lightening patterns among female students: A cross-sectional study in Saudi Arabia.** Int J Womens Dermatol 2019; 5(4), 246-250.

Ehiaghe L. Anaba, Ruth Itohan Oaku, Cole Olufolakemi Motunsope. **Hospital-based prospective study of the clinical and epidemiologic profile of adult female acne in Lagos, Nigeria.** Int J Womens Dermatol 2019; 5(4), 275-276.

In Young Chung, Celestine C. Wong, Michelle A. Rodrigues. **The importance of dermoscopy in subclinical lichen planus in skin of color.** Int J Womens Dermatol 2021; 7(2), 205-206.

Patricia F. Coogan, Traci N. Bethea, Yvette C. Cozier, Kimberly A. Bertrand, Julie R. Palmer, Lynn Rosenberg, Yolanda Lenzy. **Association of type 2 diabetes with central-scalp hair loss in a large cohort study of African American women.** Int J Women's Dermatol 2019; 5(4), 261-266.

DiAnne S. Davis, Camille Robinson, Valerie D. Callender. **Skin cancer in women of color: Epidemiology, pathogenesis and clinical manifestations.** Int J Womens Dermatol 2021; 7(2), 127-134.

Ncoza C. Dlova, Lisa O. Akintilo, Susan C. Taylor. **Prevalence of pigmentary disorders: A cross-sectional study in public hospitals in Durban, South Africa.** Int J Womens Dermatol 2019; 5(5), 345-348.

Ista A. Egbeto, Emily L. Guo, Helena B. Pasieka. **A red-orange rash in a patient with skin of color.** Int J Womens Dermatol 2021; 7(2), 203-204.

Nkechi Anne Enechukwu, Ogochukwu Ifeanyi Ezejiofor, Anaje Chetanna Chioma, Jisieike-Onuigbo Nonyelum Nnenna, Ogunbiyi Adebola Olufunmilayo, Yaria Joseph, Richard Uwakwe. **Psychosocial determinants of the practice of skin bleaching in young adult female undergraduates in Anambra State, Nigeria.** Int J Womens Dermatol 2019; 5(4), 277-278.

Ifa Etesami. **Motivations and characteristics of patients seeking minimally invasive cosmetic procedures in 2 Iranian dermatology centers.** Int J Womens Dermatol 2021; (In Press)

Ekene Ezenwa, Jennifer A. Stein, Loren Krueger. **Dermoscopic features of neoplasms in skin of color: A review.** Int J Womens Dermatol 2021; 7(2), 145-151.
S. Zahra Ghodsi, Arefeh Asadi, Narges Ghandi, Kamran Balighi, Hamidreza Mahmoudi, Robabeh Abedini, Maryam Ghiasi, Vahideh Lajevardi, Cheyda Chams-Davatchi, Maryam Daneshpazhooh. **Family impact of pemphigus disease in an Iranian population using the Family Dermatology Life Quality Index.** Int J Womens Dermatol 2020; 6(5), 409-413.

Pearl E. Grimes. **Updates in the understanding and treatments of skin and hair disorders in women of color.** Int J Womens Dermatol 2015; 1(2), 76.

Pearl E. Grimes, Melanie M. Miller. **Vitiligo: Patient stories, self-esteem, and the psychological burden of disease.** Int J Womens Dermatol 2018; 4(1), 32-37.

Pearl E. Grimes, S. Ijaz, Rama Nashawati, Daniel Kwak. **New oral and topical approaches for the treatment of melasma.** Int J Womens Dermatol 2019; 5(1), 30-36.

Aimen Ismail, Josette R. McMichael, Benjamin K. Stoff. **Utility of international store-and-forward teledermatopathology among a cohort of mostly female patients at a tertiary referral center in Afghanistan.** Int J Womens Dermatol 2018; 4(2), 83-86.

Caleb Jeon, Oma N. Agbai, Daniel C. Butler, Jenny E. Murase. **Dermatologic conditions in patients of color who are pregnant.** Int J Womens Dermatology 2017; 3(1), 30-36.

Preeti Jhorar, Reid A. Waldman, Jenna R. Bordelon, Diane L. Whitaker-Worth. **Differences in dermatology training abroad: A comparative analysis of dermatology training in the United States and in India.** Int J Womens Dermatol 2017; 3(3), 164-169.

J. Jiang, Oyindamola N. Akinseye, Andrea Paola Tovar-Garza, Amit G. Pandya. **The effect of melasma on self-esteem: A pilot study.** Int J Womens Dermatol 2018; 4(1), 38-42.

Jerome Kaikati. **The impact of acne treatment on quality of life and self-esteem: a prospective cohort study from Lebanon.** Int J Womens Dermatol 2020; 7(4), 415-421.

Kambiz Kamyab, Maryam Abdollahi, Elaheh Nezam-Eslami, Azita Nikoo, Kamran Balighi, Zahra S. Naraghi, Maryam Daneshpazhooh. **Longitudinal melanonychia in an Iranian population: a study of 96 patients**. Int J Womens Dermatol 2016; 2(2), 49-52.

Usha Khemani, Snehal Pardeshi, Anmol Sodhi. **Successful treatment of basal cell carcinoma on face and scalp by photodynamic therapy in Indian patients with dermoscopic diagnoses and serial follow-up.** Int J Womens Dermatol 2019; 5(4), 279.

Faisal Khosa. **Representation of Sex, Race and Ethnicity in Pivotal Clinical Trials for Dermatologic Drugs.** Int J Womens Dermatol 2021; 7(4), 428-434

Emily K. Kozera, Anes Yang, Dedee F. Murrell. **Patient and practitioner satisfaction with tele-dermatology including Australia's indigenous population: A systematic review of the literature.** Int J Womens Dermatol 2016; 2(3), 70-73.

Shelby L. Kubicki, Shadi Damanpour, Ranon Mann. **Ketoconazole shampoo-induced hair discoloration**. Int J Womens Dermatol 2020; 6(2), 121-122.

Vahideh Lajevardi, Robabeh Abedini, Mehdi Moghaddasi, Seyed Farzad Mirfallah Nassiri, Azadeh Goodarzi. **Bone mineral density is lower in male than female patients with plaque-type psoriasis in Iran.** Int J Womens Dermatol 2017; 3(4), 201-205.

Christina N. Lawson, Jasmine Hollinger, Sumit Sethi, Ife Rodney, Rashmi Sarkar, Ncoza Dlova, Valerie D. Callender. **Updates in the understanding and treatments of skin & hair disorders in women of color.** Int J Womens Dermatol 2017; 3(1), S21-S37.

Ida Aurélie Lenga-Loumingou. Dermatoses and traditions: **Fissured plantar keratoderma, a discriminating factor in bantu society in the Congo Brazzaville.** Int J Womens Dermatol 2020; 6(2), 125-126.

Shari Lipner. **Analysis of Skin Color in the American Academy of Dermatology Basic Dermatology Curriculum.** Int J Womens Dermatol 2021; 7(4), 505-507

Tiffany T. Mayo, Valerie D. Callender. **The art of prevention: It's too tight—Loosen up and let your hair down.** Int J Womens Dermatol 2021; 7(2), 174-179.

Uchenna R. Okereke, A. Simmons, Valerie D. Callender. **Current and emerging treatment strategies for hair loss in women of color.** Int J Womens Dermatol 2019; 5(1), 37-45.

Jadesola T. Olayinka, Mona A. Gohara, Quincy K. Ruffin. **#BlackGirlMagic: Impact of the social media movement on Black women's self esteem.** Int J Womens Dermatol 2021; 7(2), 171-173.

Ginikanwa Onyekaba, Ademide Adelekun, Susan C. Taylor, Temitayo Ogunleye. **Assessing the impact of an intervention to increase minority representation in dermatology.** Int J Womens Dermatol 2021; 7(2), 199-200.

John G. Plante, Ahmad I. Aleisa, Bruce H. Thiers. **Pityriasis rubra pilaris in skin of color.** Int J Womens Dermatol 2021; 7(2), 207-208.

Samara Pollock, Susan Taylor, Oyetewa Oyerinde, Sabrina Nurmohamed, Ncoza Dlova, Rashmi Sarkar, Hassan Galadari, Mônica Manela-Azulay, Hae Shin Chung, Evangeline Handog, A. Shadi Kourosh. **The dark side of skin lightening: An international collaboration and review of a public health issue affecting dermatology.** Int J Womens Dermatol 2021; 7(2), 158-164.

Jodie Raffi, Raagini Suresh, Oma Agbai. **Clinical recognition and management of alopecia in women of color.** Int J Womens Dermatol 2019; 5(5), 314-319.

Farzana Rahiman. **A survey evaluating knowledge, perception and use of skin lightening products among South African students.** Int J Womens Dermatol 2021; (In Press)

Sara Sabourirad, Dedee F. Murrell. **A dark lesion on an Indian Woman's vulva.** Int J Womens Dermatol 2021; 7(2), 201-202.

Lauren Seale, Olabola Awosika, Henry W. Lim. **Trends in sessions in diversity at the American Academy of Dermatology Annual Meetings: 2013–2019.** Int J Womens Dermatol 2021; 7(2), 197-198.

Sitaula Seema, Amrita Neupane, Anil Kumar Das. **White ambition leading to topical corticosteroid misuse - A beauty myth in skin of color.** Int J Womens Dermatol 2019; 5(4), 278-279.

Hannah Song, Ashley Beckles, Prerna Salian, Martina L. Porter. **Sunscreen recommendations for patients with skin of color in the popular press and in the dermatology clinic.** Int J Womens Dermatol 2021; 7(2), 165-170.

Amir Teimourpour, Kowsar Hedayat, Fereshteh Salarvand, Narges Ghandi, Maryam Ghiasi, Hamidreza Mahmoudi, Kamran Balighi, Roja Toosi, Farnam Mohebi, Ali Nili, Maryam Daneshpazhooh, Dedee F. Murrell, Cheyda Chams-Davatchi. **Autoimmune Bullous Disease Quality of Life (ABQoL) questionnaire: Validation of the translated Persian version in pemphigus vulgaris.** Int J Womens Dermatol 2020; 6(4), 306-310.

Herbert B. Castillo Valladares, Alison K. Lee, Shayan Cheraghlou, Amanda Zhou, Sarika Ramachandran. **A pilot study examining skin cancer education in an underserved population at a free skin cancer screening.** Int J Womens Dermatol 2021; 7(2), 184-186.

Britney N. Wilson, Jenny E. Murase, Diane Sliwka, Nina Botto. **Bridging racial differences in the clinical encounter: How implicit bias and stereotype threat contribute to health care disparities in the dermatology clinic.** Int Journal Womens Dermatol 2021; 7(2), 139-144.

Britney N. Wilson, Mary Sun, Alyssa Gwen Ashbaugh, Simran Ohri, Christopher Yeh, Dedee F. Murrell, Jenny E. Murase. **Assessment of skin of color and diversity and inclusion content of dermatologic published literature: An analysis and call to action.** Int J Womens Dermatol 2021; 7(4), 391-397.

Lauren E. Wiznia, Jenny Wang, Alexa B. Steuer, Nada Elbuluk. **Direct-to-consumer dermatology-related advertising differs in magazines targeted to women of color: A cross-sectional analysis of top-circulating consumer magazines.** Int J Womens Dermatol 2021; 7(2), 191-194.

Mukhtar A. Yusuf, Bobbi S. Pritt, Josette R. McMichael. **Cutaneous myiasis in an elderly woman in Somaliland.** Int J Womens Dermatol 2019; 5(3), 187-189.

Mukhtar A. Yusuf, Nicma D. Mahmoud, Farhan R. Rirash, Benjamin K. Stoff, Yuan Liu, Josette R. McMichael. **Skin lightening practices, beliefs, and self-reported adverse effects among female health science students in Borama, Somaliland: A cross-sectional survey.** Int J Womens Dermatol 2019; 5(5), 349-355.

### Gender minority and transgender diversity in our literature

The focus of the IJWD is on the dermatologic health of women and their families. This range of topics includes but is not limited to the role of hormones in the development of acne and alopecia; the skin manifestation of metabolic syndrome and polycystic ovarian syndrome; skin cancer risk specific to women; clinical considerations when managing chronic dermatologic conditions (e.g., atopic dermatitis, psoriasis, and cutaneous lupus in women); the safety profile of dermatologic medical and cosmetic therapy in pregnant and breastfeeding women; the impact of esthetic procedures on body image and body dysmorphic disorders; chemotherapeutic concerns in dermatologic skin care in women with cancer; skin disease specific to women who are aging in menopause, pregnant, or breastfeeding; skin disease of the vulva and vaginal area; vaginal rejuvenation; and ethical issues when caring for dermatologic issues of women and their families. But importantly it also includes issues for our colleagues and patients with issues regarding gender minority and transgender dermatology, as well as gender minority and sexual orientation bias in the workplace. We have published articles related to transgender and gender minority patients (Table 5), and this area in particular is one that we wish to focus our attention on moving forward and to purposefully recruit articles to further our strategic initiative as we strive to develop a culture of inclusion in dermatologic publishing.

**Table 5:** Articles published in support of dermatologic health concerns for our **gender minority and transgender** patients

Brian A. Ginsberg. **Dermatologic care of the transgender patient**. Int J Womens Dermatol 2017; 3(1): 65-67.

Shankar N. Mundluru, Joshua D. Safer, Allison R. Larson. **Unforeseen ethical challenges for isotretinoin treatment in transgender patients**. Int J Womens Dermatol 2016; 2(2): 46-48.

Shankar N. Mundluru, Allison Ruth Larson. **Medical dermatologic conditions in transgender women**. Int J Womens Dermatol 2018; 4(4): 212-215.

Kyla N. Price, Afsaneh Alavi, Jennifer L. Hsiao, Vivian Y. Shi. **Gender minority patients in dermatology clinical trials.** Int J Womens Dermatol 2020; 6(5): 438-439.

### Creation of culture of inclusion in dermatologic publishing

Ultimately, we believe editors, editorial boards, and publishers should be encouraged to critically examine diversity in their publications, including an annual assessment with metrics indicating how effectively a culture of inclusion has been fostered. Currently, journal success is evaluated through bibliometric milestones, such as Scopus scores, number of submissions and citations, and impact factors, but the culture of inclusion that the journal has built should also be factored into an evaluation of a journal's impact on its subspecialty and the importance of that journal in the field of medicine and within society.

We encourage evaluation metrics that

1) Examine editorial board and advisory board make up, including race, gender, ethnic background, and country of origin, the latter particularly pertaining to international journals.
2) Examine how the publication supports professionals from different backgrounds.
3) Examine the gender, race, and ethnic background of its authorship.
4) Examine topics published to foster knowledge of gender differences, skin of color content, sexual minority, gender minority and transgender issues, when appropriate.

It is ultimately the development of a culture of inclusion, a strategic initiative that is purposefully and thoughtfully executed by editors and editorial board members, that brings change to our society. Editors have a great responsibility in deciding what information is disseminated to medical professionals and the public in a peer-reviewed platform, where potentially more weight is placed on the validity of the findings published due to the rigorous review process for the published articles. It is easy to state that the inequity in our literature is not being addressed by the stream of articles submitted to the journal, but what if, as editors, we take the initiative to invite publications that directly address this inequity in our literature? Or to encourage research that would address these inequities? Or to welcome voices that would otherwise be unheard? The best counter action to discrimination against populations that have been marginalized in the past is to be intentional in preventing that discrimination from continuing. The onus is on us as journal editors to create a welcoming and inclusive environment that embraces our differences and is enriched by the knowledge and insight that results when these differences are identified, classified, analyzed, and studied. Diversity is important, but the development of a culture of inclusion on our editorial boards, in our authorship, and within our publication is the key to unlocking new material that benefits both the majority and the minority, the male and the female, the mainstream and the marginalized. Encouraging the voices of the marginalized is the essence of what allows for the creation of this culture. Therefore, fostering a culture of inclusion is vital to gaining access to the benefits of diversity. Let the marginalized of the past be heard for all of us to reap the benefits of inclusion.
